# Continuing medical education revisited: theoretical assumptions and practical implications: a qualitative study

**DOI:** 10.1186/s12909-014-0278-x

**Published:** 2014-12-31

**Authors:** Alexander Dionyssopoulos, Thanassis Karalis, Eugenia A Panitsides

**Affiliations:** Department of Plastic Surgery, Faculty of Medicine, Aristotle University of Thessaloniki, Periferiaki Odos Thessalonikis, 56403 Thessaloniki, Greece; Department of Educational Science and Early Childhood Education, University of Patras, Campus GR-26504, Rio, Achaia, Greece; Department of Educational and Social Policy, University of Macedonia, Thessalonikim, Greece

**Keywords:** Continuing medical education, Adult learning, Learner-centered approach, Group interaction, Plastic surgery, Qualitative research

## Abstract

**Background:**

Recent research has evidenced that although investment in Continuing Medical Education (CME), both in terms of participation as well as financial resources allocated to it, has been steadily increasing to catch up with accelerating advances in health information and technology, effectiveness of CME is reported to be rather limited. Poor and disproportional returns can be attributed to failure of CME courses to address and stimulate an adult audience.

**Methods:**

The present study initially drew on research findings and adult learning theories, providing the basis for comprehending adult learning, while entailing practical implications on fostering effectiveness in the design and delivery of CME. On a second level, a qualitative study was conducted with the aim to elucidate parameters accounting for effectiveness in educational interventions. Qualitative data was retrieved through 12 in-depth interviews, conducted with a random sample of participants in the 26^th^ European Workshop of Advanced Plastic Surgery (EWAPS). The data underwent a three level qualitative analysis, following the “grounded theory” methodology, comprising ‘open coding’, ‘axial coding’ and ‘selective coding’.

**Results:**

Findings from the EWAPS study come in line with relevant literature, entailing significant implications for the necessity to apply a more effective and efficient paradigm in the design and delivery of educational interventions, advocating for implementing learner-centered schemata in CME and benefiting from a model that draws on the learning environment and social aspects of learning.

**Conclusions:**

What emerged as a pivotal parameter in designing educational interventions is to focus on small group educational events which could provide a supportive friendly context, enhance motivation through learner-centered approaches and allow interaction, experimentation and critical reflection. It should be outlined however that further research is required as the present study is limited in scope, having dealt with a limited sample.

## Background

Adult learning is not a new concept. It has been encountered as early as in the works of Plato and Aristotle (Plato’s Republic; Aristotle Politics), with ‘paideia’ throughout life being considered the ‘grandiose and sole means’ for harmonious self development and fulfillment. Yet, what has gained adult education and training a new momentum as an overarching policy goal is the development of the Human Capital Theory (HCT) during the 1960s, grounded on the assumption that investment in human resources, namely on the development of individuals’ skills and knowledge, generates economic benefits for economies, organizations, individuals and society as a whole [1,2]. HCT has widely influenced education and training policies throughout Western countries, giving rise to professional development schemata. Human Resource Development (HRD), as a set of systematic and planned interventions providing individuals with the opportunity to learn necessary skills to meet current and future job professional demands [3], has become a priority in the contemporary knowledge based and technology driven context, in which (re)skilling has turned into the norm to meet changing professional requirements.

In this regard, Continuing Medical Education (CME) has been propounded as an indispensible means for catching up with accelerating advances in health science and technology, as well as for improving medical performance, while financial resources allocated to it have been steadily increasing. More precisely, acknowledging that medical personnel is employed in a most pertinent working field, the impetus for flexibility, adaptability and improved performance has come to the fore. Throughout the contemporary literature [4-6], the necessity for bringing physicians up-to-date with an evolving body of knowledge, so as to improve performance and optimize outcomes, is emphasized, while delivering CME interventions has become a major enterprise, globally [4,7]^.^

However, there has been conflicting evidence regarding returns of investment in CME, both in terms of financial resources allocated and time invested (physicians report spending on average 50 hours per year), in medical performance and patient outcomes [4-6,8-11]. According to relevant studies [5,8,9,11], limited effectiveness of CME as a performance improvement tool can be attributed to the fact that most interventions are patterned after undergraduate medical education, focusing primarily on an “educational” rather than an “occupational” model, carried out by traditional methods, such as lectures, presentations and enduring materials, through teacher-centered approaches, failing thus to stimulate an adult audience. Moreover, major emphasis of CME has been put on accruing credentials and qualifications rather than improving actual performance [5].

It has therefore become a mandate to apply more effective and efficient paradigms in the delivery of CME interventions, informed by adult education theoretical framework, while following a performance-oriented schema [12,13]. In this respect, the present study initially drew on research findings and adult learning theories, entailing practical implications on the design and delivery of CME. On a second level, a case study was conducted with the aim to further elucidate parameters associated with the effectiveness of educational interventions.

### Enhancing adult learning: Theoretical assumptions and practical implications

#### I hear and I forget. I see and I remember. I do and I understand (Confucius, 551–479 BC)

Since antiquity, it has been presumed that education and learning are social processes, stressing the fundamental role of social interaction while denoting the importance of experience in cognitive development. Yet, only recently psycho-cognitive research has elucidated some of the mechanisms accounting for human learning, designating specific critical parameters with reference to the educational context.

There is sufficient evidence to suggest that certain factors are able to promote neuroplasticity, namely the ability of the human brain to change its mental representations in response to environmental stimulation [14]. Neuronal branching can actually be stimulated by a stimuli-rich, “secure” learning context characterized by collaboration and mutual support, eliciting motivation and providing for contextually embedded information [15,16]. Furthermore, mirror neural networks [17,18] have been found to play a significant “empathic” role in social behavior and interpersonal relationships, accounting for the affective tone within a group. Hence, group cohesion in CME interventions emerges even before verbal interaction, in the sense that participants feel more connected and willing to cooperate and strive for a common goal, confirming the social nature of learning. Finally, the amygdala is considered to be involved in regulating memory consolidation of emotionally arousing experiences [19-21], determined by the extent of the emotional response an event invokes. In other words, emotionally significant information is encoded and can be easier retrieved, signifying that we tend better to learn and remember those things that entail added emotional emphases, while we have to work harder at recall to retrieve information that has no special meaning [22,23].

Research findings in cognitive neuroscience and psychology have merely evidenced what in fact has been empirically known since the works of ancient Greek philosophers (the Socratic teaching method, Plato’s holistic approach to education and the Aristotelian “learn by the actual doing”), until Vygotsky's [24] and Bandura’s [25] socially embedded theories, as well as the empirically based theory of knowledge developed by Dewey [26].

In this context, a most influential theory in the field of adult learning, having built on the works of Dewey, is “Experiential Learning” theory. Kolb [27,28] outlined that experience plays a pivotal role in the learning process, whereby knowledge is created through the transformation of experience, following a cyclical process comprising four stages: concrete experience, reflective observation, abstract conceptualization and active experimentation. As indicated in Kolb’s model, a critical parameter integral to processing experience so as to promote cognitive development, is critical reflection. In effect, in light of the transformative learning theory [29,30], reflection on experience and rational-reflective discourse may lead to perspective transformation, while in order to engage in reflective discourse presupposes the ability to examine alternative perspectives, involving a critical assessment of assumptions and reaching a clearer understanding of experience to arrive at a tentative best judgment [31]. Recent studies on transformative learning have also emphasized the importance of relationships in the learning process [32], while a central finding of research over the past 15 years is that a key to developing an integrated and generative knowledge base is to build upon the learners’ prior knowledge, as for something to be learned it has to fit into the learner’s established knowledge network [33].

Experience, thus, has long been a cornerstone in learning, especially when infused with reflective thought [34] and building upon prior knowledge. Thus, information which is contextually embedded is easier to learn, as the brain is not skilled in learning isolated sequential bits of information, yet quick to learn in situations that are closer to reality, while it is stimulated by positive emotions and multiple sensory experiences [16,22,35]. More importantly, however, beyond contextualization, experiencing and reflection, learning has a prevailing social dimension which has been widely acknowledged as an important perspective for understanding the interrelations between learning, social contexts and interpersonal interactions [25,26,36]. This also comes in line with the social constructivist learning theory which asserts that construct of knowledge takes place through a process of experience sharing and interactive discussion [37].

Studies on group dynamics in CME have also indicated the psychosocial and affective dimension of educational interventions as a central plank in effective learning [38-42]. More precisely, to facilitate learners’ movement from the isolation stage to the bonding stage, it is deemed necessary to build a safe and open learning environment, in which participants’ diversity and experiences are respected and valued, and group identity and mutual commitment are gradually developed [42]. Anti-productive teams tend to function in a defensive manner, with participants confronting anxiety and lack of trust. Conversely, a productive team is open to conflict, allowing learners to interact, freely express, sometimes controversial, thoughts and beliefs, as well as share interests and experiences [43,44].

Pereles, Lockyer and Fidler [38], investigating the role of small groups as part of the social structure provision in which learning can take place, concluded that they serve as an ideal setting for participants to freely listen, reflect and share opinions and clinical experience. In the same vein, Wenger [39] argued that trust among learners is the most critical parameter in educational interventions, accounting for the commitment of participants in the learning process, the effective tackling of challenges and the widening of learning objectives and methods.

It is evident from the above brief discussion of some studies that, in reframing CME interventions to cater for improved learning outcomes, it is critical to foster an experiential and interactive educational context which is likely to contribute to reinforcing participants’ motivation and participation, promoting their cognitive and competence development [41,45-47]. In order to shed further light on the issue under discussion, a qualitative study was carried out with a sample of Plastic Surgeons with the aim to further elucidate parameters accounting for effectiveness in educational interventions.

## Methods

An exploratory study was conducted, on the basis of participants’ perceived views and experiences of engagement in a learner-centered interactive educational event. The aims of the study were twofold, translated into research questions, which guided the procedures and practices throughout the study:

(1) To investigate and identify factors that may account for promoting motivation and learning during CME interventions

(2) To set a basis for grounded suggestions on enhancing effectiveness of CME interventions, as well as to provide signposts for further research.

In conducting the study, the qualitative approach was followed, with a view to obtaining an in-depth insight into underlying processes and interrelations among events and situations [48,49]. In selecting a case from which the most could be learned [48], the case of the European Workshop of Advanced Plastic Surgery (EWAPS) [50] was selected. EWAPS was established in 1986 after the initiative of five plastic surgeons that formed a forum to discuss clinical and scientific advancements. It actually functions as a “community of practice”, namely a self-organized selected group of individuals who share common interests as well as a common sense of purpose and a desire to learn from each other [39]. Thus, it serves as an ideal case in investigating parameters associated with learner-centered approaches in an interactive learning context.

In an attempt to gain meaningful insights into the situation, qualitative data were obtained through 12 in-depth interviews, which were conducted with a random sample. Randomization of the interviewees was performed by selecting individuals listed under even numbers from the 25 enlisted participants in the 2012 EWAPS meeting. Participants’ consent was obtained after being duly informed on the purpose of the study and the interview process, while it was explicitly stated that participation was entirely voluntary and that their anonymity would be ensured. Ethical clearance was obtained from the Faculty of Medicine of the Aristotle University of Thessaloniki Bioethics Committee (reference number: 108/14-11-14).

The interviews were carried out using a research tool based on detailed field notes from participant observation [48] during three consecutive meetings (2009–2011), which were used as a blueprint in building the interview main axes: group relationships, learner-centeredness, active engagement, critical reflection, cognitive and competence development, performance improvement. All interviews were conducted in person in a relaxed atmosphere, whereby the participants were encouraged by the interviewer, a researcher in the field of adult education, to freely express their ideas, through open-ended questions prompting spontaneous information.

The transcribed data underwent a three level qualitative analysis, following the “grounded theory” methodology [51], comprising ‘open coding’, ‘axial coding’ and ‘selective coding’. In ‘open coding’, a constant comparative analysis was used to develop descriptive codes [52], conceptualizing and categorizing the interview data. At the ‘axial coding’ stage, whereby data were put back together in new ways by utilizing the coding paradigm involving ‘conditions, context, action/interaction strategies and consequences’ [51], an attempt was made to identify and define causal connections between categories. Finally, through ‘selective coding’, the core variable was selected, systematically relating it to other categories, validating relationships and filling in categories that needed further refinement and development.

## Results and discussion

EWAPS meetings take place annually, while participation of representatives from as many European countries as possible is encouraged. The profile of participants in the workshops, held from 2009 to 2012, is depicted in Table 1.

**Table 1:** **Profile of participants in the EWAPS 2009–12 workshops**

**Participants**		**Count**	**%**	**Total**
	*Participating members*	35	72.9	48
	*Invited participants*	13	27.1	
**Gender**	*Male*	38	79.2	48
	*Female*	10	20.8	
**Country of origin**	*Austria*	4	8.3	48
	*Belgiun*	1	2.1	
	*France*	5	10.4	
	*Germany*	7	14.6	
	*Greece*	4	8.3	
	*Italy*	4	8.3	
	*Norway*	4	8.3	
	*Poland*	1	2.1	
	*Slovenia*	1	2.1	
	*Spain*	4	8.3	
	*Sweden*	4	8.3	
	*Switzerland*	6	12.5	
	*United Kingdom*	3	6.3	
**Experience in Plastic Surgery**	*Specialist over 5 years*	6	12.5	48
	*Specialist over 10 years*	9	18.8	
	*Specialist over 15 years*	33	68.7	

Out of the twelve interviewees, having been randomly selected from participants in the 26^th^ EWAPS annual workshop, the majority were men (8), experienced surgeons with an experience exceeding 15 years (7), mainly in private practice (8). Through comparative analysis, conceptualizing and categorizing the transcribed interview data, twenty-one descriptive codes/labels [52] emerged, which are presented in Table 2.

**Table 2:** **Open coding of data**

**Labels**	**Interviewees**	**M ≥15yPP**	**M ≥15y PP**	**F ≥15yPP**	**F ≥15yPP**	**M ≥15ySH**	**M ≥15ySH**	**M ≥15ySH**	**F ≥5y, PP**	**M ≥15ySH**	**M ≥5y, PP**	**M ≥10yPP**	**F ≥5yPP**	**Total**
Knowledge_Skills_Improvement		1	1	1	1	1	1	1		1		1	1	**10**
Experiential_Learning		1	1	1			1	1	1	1	1		1	**9**
Minimal_Lecturing		1	1	1		1			1		1		1	**7**
Problem-based_Learning				1	1	1	1			1		1	1	**7**
Creative_Interaction			1		1			1	1		1		1	**6**
Critical_Reflection				1				1	1	1		1	1	**6**
Experience_Sharing				1	1					1	1	1	1	**6**
Participation_Criteria		1		1	1	1	1			1				**6**
Peer_Learning					1			1	1	1	1	1		**6**
Collaborative_Context			1		1					1	1	1		**5**
Creative_ Feedback		1	1			1	1	1						**5**
Friendly_Context					1	1		1	1		1			**5**
Active_Participation		1			1	1								**3**
Casual_Atmosphere					1		1				1			**3**
Family_Friendly		1		1									1	**3**
Fruitful_Discussion		1	1	1										**3**
Knowledge_Sharing							1	1	1					**3**
Practice_Improvement			1			1			1					**3**
Prior_Knoweledge_Stimulation							1			1		1		**3**
Practice_Oriented						1					1			**2**
Socializing									1		1			**2**

*M = Male, F = Female, Specialist ≥ n years, PP = Private Practice, SH = State Hospital.

The interviewees’ experience in Plastic surgery and the gender factor did not seem to have any impact on the outcomes of the study. By comparing the labels that emerged through ‘open coding’, three categories were identified that were congruent, mutually exclusive, and exhaustive [51]: “closed training event”, “problem-based learning” and “skills workshops” (Table 3).

**Table 3:** **Axial and selective coding of data**

**Core category**	**Categories**	**Labels**
**Friendly-Collaborative context**	Closed training event	*Safe supportive environment*
		*Peer learning*
		*Interactive context*
	Problem-based learning	*Activating prior learning*
		*Critical reflection*
		*Feedback provision*
		*Creative learning*
	Skills Workshops	*Experiential learning*
		*Active involvement*
		*Competence-based learning*

It is important to note that all interviewees had a positive attitude towards perceived returns of their participation in the EWAPS workshops, acknowledging that “valuable” knowledge, skills and competences were acquired, which would enable them to improve their practice.

Among parameters covered by interviewees to be accounting for the EWAPS positive atmosphere was the fact that annual workshops have been designed as closed events and the attendance is restricted to members. A prerequisite for a non member to participate is by invitation of the board of participants only, on the premise that he/she fulfills certain criteria. Moreover, it was noted that the scientific program is counterbalanced by a variety of social activities which include all participants and their families. This policy has been considered by interviewees to contribute to establishing over the years a friendly collaborative atmosphere amongst members, which even exceeds the remit of the annual meetings. As a female participant reported:

*“You see, they are not just colleagues. They are friends. I can share thoughts and problems, I couldn’t talk about otherwise. And this doesn’t stop here… We keep in touch and collaborate all year round …”.*

Another critical parameter outlined by the majority of interviewees was the fact that each presentation should not normally exceed three minutes, followed by a twelve minute discussion. The specific practice is considered to have considerably reduced the lecture-centered approach, allowing sufficient time for prompting dialogue and promoting critical reflection and interaction. An experienced male participant noted:

*“I have been to hundreds of meetings and workshops all over the world. Yet, most times they just bore me … all that often pointless talking… In EWAPS I have found a community to share knowledge and experience, while discuss and reflect on issues of shared interest”.*

Furthermore, presentations tend to follow a problem-based approach [45], falling under five thematic axes: *Cry for help*, *My original technique*, *My worst experience*, *Ideas and Innovations* and *Follow up*. Among the five axes, the *Cry for help* and *My worst experience* sections have been indicated to substantially promote interaction within the group, while recalling of prior knowledge was reported to be stimulated and critical reflection to be fostered. Moreover, these particular workshops were reported to have reinforced the sharing of accumulated knowledge and experience, along with prompting creative thinking in order to provide for solutions to the problems posed. As reported by an experienced surgeon with regard to the “Cry for help” workshop:

*“This year I have brought to discussion two cases that really troubled me. A lot of brainstorming took place and got valuable feedback from all colleagues here… I can’t wait to get back and try out the solutions we reached”.*

Finally, skills workshops taking place in the context of each meeting were indicated to enhance experiential learning. In the 2012 meeting, a hands-on workshop was organized with live models on new fillers and innovative non-surgical face-lifting techniques. All interviewees acknowledged to have provided an ideal context for active learning. Peer-learning was also outlined to have taken place, pointing out that knowledge-sharing did not follow a linear mode – from older to younger surgeons – but the younger had some innovative techniques to share as well. A younger participant was proud to report that he had presented an innovative technique that attracted the interest of more experienced participants:

*“They all came over after the workshop, asking for further details… It’s my first time here but I think I will keep on attending the EWAPS meetings. It’s nice to be able to share both strengths and weaknesses with your peers…”.*

**Figure 1:**
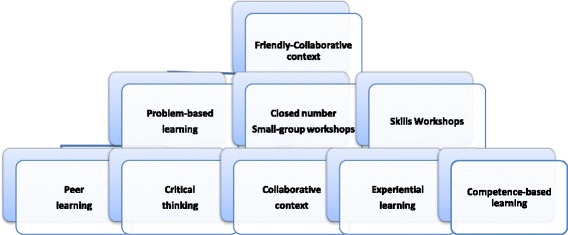
**The EWAPS educational scheme.**

Applying the coding paradigm [51], a further attempt was made to trace causal relationships among categories and reach grounded conclusions (Figure 1). Hence, through selective coding, “friendly collaborative context” was identified as the core category, being systematically related to other categories. More precisely, the friendly, trustful, non-threatening to the participants’ ego, context has been assumed to be intertwined with the collaborative, knowledge sharing approach fostered through the meetings and to reinforce learning outcomes. Participants reported that they felt comfortable when received feedback and were willing to discuss their deficiencies and reflect on them, often transforming both perspective and practice. On the contrary, in an austere context, participants admitted that it would have been difficult to substantially interact and share experiences, weaknesses and problems faced in their everyday practice, or cooperate to find answers and reach solutions.

Indeed, the need to draw on motivating educational contexts, in which the learning responsibility is passed on to the participants, relying on learner-centered approaches rather than being restricted to lectures [45,46], has been increasingly emphasized at all levels of the educational continuum, including CME. More importantly though, it should be highlighted that all educational interventions should foster an encouraging learning environment apt to promote active involvement, experimentation, interpersonal interaction and collaboration [37,46]. This should take into account that emotions and sensory experiences are integrally involved in the learning process [35].

Our results were in line with other relevant research findings [5,6,8-11,38-44,47] suggesting that CME interventions may be more effective when they cater for small-group cooperative (peer) learning based on case studies and clinical scenarios, as well as on skills workshops and simulations. Furthermore, qualitative analysis of the data indicated that a significant parameter in designing educational interventions is to focus on educational events which could provide a supportive friendly context, enhance motivation through learner-centered approaches and allow interaction, experimentation and critical reflection. All these factors were identified by interviewees as critical, accounting for EWAPS’ potential impact on clinical performance. In fact, our findings suggested that the small group setting is an ideal forum for maintaining a sense of “community” and for fostering mutual sharing and understanding, with the mentoring role [53] being highly valued.

In this context, it could be argued that a great deal of CME deficiencies identified in relevant studies [4,8], such as the tendency of clinicians to gravitate towards topics they are already familiar with and avoid areas they lack experience, might be attributed to failure in fostering a collaborative setting, apt to promote emotional security and facilitate personal exposure. Considering that doctors are indulged in a “culture of excellence”, first as students and then as members of a professional elite group, one could argue that it may be embarrassing for them to admit cognitive or competence deficiencies, unless they are in a friendly supportive context.

As felt and expressed by participants in the study, it would be difficult for them to freely interact and share knowledge, experience and problems faced in their practice, or to seek cooperation to find solutions, in a traditional “austere” context. Most probably, workshops would be confined to presentations followed by a set of typical questions, which is usually the case in almost all traditionally structured CME interventions [5,8,9,11].

## Conclusions

The present study has first of all drawn on earlier research findings and adult learning theories, which provided the basis for comprehending adult learning, while entailing practical implications in fostering the effectiveness of the design and delivery of CME. At the next stage, a qualitative in-depth study was conducted with the aim of delving into the parameters accounting for effectiveness in educational settings.

The findings from the EWAPS case study have shed some light on the importance of certain factors, with a major emphasis on the psychosocial and affective dimensions, which educational interventions could benefit in improving learning outcomes and increasing returns of investment in CME. The implementation of learner-centered schemata in particular that draw on the learning environment and social aspects of learning, seems to account for large part of CME effectiveness, while the structure of the interventions seems to be equally critical as well. In the EWAPS case, the workshops were structured in a way that limits lecturing and fosters interaction and problem-based learning.

The results of the EWAPS study are congruent with previous findings, entailing significant implications for the design and delivery of CME interventions. It should be noted however that the present study, besides the self-reported nature of answers, is limited in scope since it has dealt with a limited sample. Therefore, further research is suggested to ascertain whether or not the present findings can be sustained. It should be kept in mind that there might be differences between various interventions, depending on the target group, the clinical area, the aim and objectives. It should also be taken into consideration that the duration of the educational events, resources available and the context are likely to play a major role in the study and its findings. Finally, the promise of CME to improve patient outcomes has to be further investigated in future research, delving into the interrelationship between education events and practice improvement.
